# MCCA-VNet: A Vit-Based Deep Learning Approach for Micro-Expression Recognition Based on Facial Coding

**DOI:** 10.3390/s24237549

**Published:** 2024-11-26

**Authors:** Dehao Zhang, Tao Zhang, Haijiang Sun, Yanhui Tang, Qiaoyuan Liu

**Affiliations:** 1 Changchun Institute of Optics, Fine Mechanics and Physics, Chinese Academy of Sciences, Changchun 130033, China; zhangdehao@ciomp.ac.cn (D.Z.); tangyanhui@ciomp.ac.cn (Y.T.); liuqy@ciomp.ac.cn (Q.L.); 2 University of Chinese Academy of Sciences, Beijing 100049, China

**Keywords:** micro-expression, optical flow method, facial coding, MCCA-VNET, vision transformer

## Abstract

In terms of facial expressions, micro-expressions are more realistic than macro-expressions and provide more valuable information, which can be widely used in psychological counseling and clinical diagnosis. In the past few years, deep learning methods based on optical flow and Transformer have achieved excellent results in this field, but most of the current algorithms are mainly concentrated on establishing a serialized token through the self-attention model, and they do not take into account the spatial relationship between facial landmarks. For the locality and changes in the micro-facial conditions themselves, we propose the deep learning model MCCA-VNET on the basis of Transformer. We effectively extract the changing features as the input of the model, fusing channel attention and spatial attention into Vision Transformer to capture correlations between features in different dimensions, which enhances the accuracy of the identification of micro-expressions. In order to verify the effectiveness of the algorithm mentioned, we conduct experimental testing in the SAMM, CAS (ME) II, and SMIC datasets and compared the results with other former best algorithms. Our algorithms can improve the UF1 score and UAR score to, respectively, 0.8676 and 0.8622 for the composite dataset, and they are better than other algorithms on multiple indicators, achieving the best comprehensive performance.

## 1. Introduction

Facial expressions, as one of the main ways in which people show their emotions, have seen important advances in research on the recognition of various expressions over the past few decades [1,2,3,4,5,6]. In these years, micro-expression recognition has gradually become a new research hotspot [7,8], and micro-expressions are often produced when a person wants to suppress his or her feelings, which are involuntarily produced and cannot be suppressed. Micro-expressions as an important basis for judging human subjective emotions, in the field of psychological treatment, criminal investigation, security, and other fields, have a wide range of applications and research; however, the training of micro-expression recognition-related professionals requires a lot of time and financial resources, so the study of micro-expression recognition is of great practical significance and far-reaching impact.

To realize the micro-expression recognition detection task on computer, according to the characteristics of micro-expression recognition, the mainstream methods are the Optical Flow (Optical Flow) method, Local Binary Pattern (LBP) [9], and Convolutional Neural Networks (CNN). Currently, micro-expression recognition algorithms are basically implemented based on these three algorithms and their improvements. Micro-expression recognition can be broken down into two steps: one is image preprocessing, and the other is micro-expression classification. Preprocessing mainly includes face detection and alignment, face cutting and image normalization. The most important breakthrough in traditional face detection techniques is the successful design of the Viola–Jones face detector. In 2001, Paul Viola & Michael Jones [10] designed an efficient face detector based on Haar features, the VJ face detector. Its algorithmic innovations include three aspects: the use of an integral map as a fast computation method for features, the use of AdaBoost as an effective classifier learning method, and an efficient cascade structure as a classification strategy. Traditional feature extraction methods are mainly categorized into gradient-based, motion-based, and texture-based feature extraction methods; Shreve [11] proposed a localization method for micro-expressions, based on the effect of non-rigid muscle movement on the skin caused by expression, using the center difference method to obtain the dense optical flow field in the chin, mouth, cheeks, and forehead regions and establish the strain magnitude coefficients and differentiating between micro-expressions and ordinary expressions based on the strain coefficients. Zhao et al. [12] utilized 68 facial landmarks and ASM (the active shape model) for face alignment and cropping, and, in order to solve the problem of a varying number of frames in different image sequences, TIM (the temporal interpolation model) was introduced for data augmentation, and LBP-TOP (Local Binary Pattern histories from Three Orthogonal Plans) feature descriptors were proposed. Wang et al. [13] optimized LBP-SIP (Local Binary Pattern with Six Intersection Points) features based on LBP-TOP and extracted dynamic texture features. Based on LBP features, shape and motion features are usually extracted from the sign-based differences between two pixels, without considering other information. Zhao et al. [14] improved this by proposing STCLQP (Spatiotemporal Completed Local Quantization Patterns) features that include sign, amplitude, and direction. STCLQP uses a 24-point local region to complete the LBP feature extraction and obtains gradient direction and magnitude features by using a Seibel operator and a Gaussian kernel, in the shape and time domains, and LBP features vector quantization and proposed an effective codebook selection. In terms of the optical flow method, after extracting the optical flow features of the micro-expression start and apex frames by the TV-L1 optical flow method, micro-expression classification has also been applied to algorithm improvement by the classical deep learning models VGG16 [15], GoogleNet [16], AlexNet [17], and OFF-Apex [18], after which micro-expression recognition via deep learning has also gradually become mainstream. Since then, different later deep learning algorithms have gradually been applied to micro-expression recognition. Zhou et al. [19] introduced an attention mechanism and proposed a method for facial expression recognition using a feature refinement network, a specific expression feature learning network, and fusion technology. By using the self-attention mechanism of global image features, significant features for micro-expression recognition were extracted, which improved the recognition rate of micro-expressions.

In the field of facial expression recognition, due to the subtle and nuanced nature of facial actions, it is difficult to extract specific facial regions and temporal micro-expression features related to micro-expressions. At the same time, current deep learning methods only consider the relationship between global and local information at the feature level, without taking into account the inherent spatial relationship between local facial regions, which can lead to poor performance in micro-expression recognition tasks. To address these issues, we propose a new approach to micro-expression recognition called MCCA-VNet. It operates as follows: utilizing the Vit layer in order to efficiently capture interactions within the local and global hierarchies, the network is able to fuse temporal and spatial features to predict classification scores; visual Transformer correlation model, Xcit-ViT, is applied in the field of micro-expression recognition and improved to achieve efficient recognition results; a hybrid attention module is incorporated that fuses channel attention and spatial attention to make the module more focused on important micro-expression factors. Our contributions in this study can be summarized as follows:

- Our network pays special attention to five facial regions—left eye region, left lip region, nose region, right eye region, and right lip region—to realize the feature extraction and fusion of key regions related to micro-expressions.
- A new self-attention mechanism of the Vit layer is introduced, which establishes a self-attention mechanism among multi-channel features, aggregates spatial attention and channel attention, and also establishes an attention mechanism for pixel dependencies among different regions, through which the refined features and temporal features of micro-expressions are captured.
- For the characteristics of micro-expression recognition, our model adjusted and improved the network structure of the VIT framework. It makes it more adapted to the micro-expression recognition task based on optical flow features and further improves the performance of the algorithm.

## 2. Related Work

There are two main categories of micro-expression recognition methods: those based on traditional manual features and those based on deep learning.

### 2.1. Manual Characteristics

Manual feature extraction methods capture the unique features of micro-expressions by manually designing visual descriptors, which are then fed into a classifier for recognition. Pfister et al. [20] first implemented micro-expression recognition using LBP and machine learning algorithms. In order to accurately process facial micro-expression features, Liu et al. [21] proposed a principal direction averaged optical flow feature extraction method, which consists of extracting and averaging the optical flow from a sequence of micro-expression directions. Some researchers recognize different classes of micro-expressions with the help of Gabor filtering and a support vector machine [22]. Ma et al. [23] introduced region histogram of oriented optical flow (RHOOF), extracted HOOF features from neighboring frames to detect apex frames, and used RHOOF features between the apex frame and the start frame to improve the recognition rate. Geometry-based features are usually categorized into two types: features based on optical flow fields and features based on texture variations. These geometry-based features can recognize motion deformations. Li et al. [24] proposed a deep learning method for localizing facial feature points and contacting facial micro-expressions and facial muscles. Using this technique, they divided the face into different muscle movement units and reflected facial muscle movements through optical flow, thereby mapping micro-expression changes. Liu proposed a feature extraction network based on MDMO, which calculates the average optical flow field features of each image in a video sequence and uses an SVM classifier to classify the obtained average optical flow field, achieving emotion recognition. Their approach effectively takes into account geographic location and region statistical motion information and proves to be simple and effective. Instead of using LBP histograms, Liong et al. [25] proposed a method for recognizing facial expressions (Bi WOOF) by integrating LBP histogram weighting and average optical flow feature values. Wang et al. [26] proposed a dynamic texture method based on tensor-independent color space for micro-expression recognition. The encoding model (DTCM) normalizes facial features while extracting effective features and eliminates invalid features to effectively encode texture changes related to facial unit activity, improving the recognition accuracy of micro-expression recognition. A series of effective experiments were conducted on the main dataset.

The micro-expression recognition method based on manually constructed features has a strong theoretical foundation and low dependence on dataset size; however, hand-constructed feature extraction usually involves the use of expert-designed extractors, a process that often requires specialized knowledge and cannot be easily adjusted to optimize the training process.

### 2.2. Deep Learning Methods

In recent years, a series of deep learning techniques have been applied to the field of micro-expression detection. Deep learning methods generally use neural networks, which can realize the end-to-end automatic extraction of deep features and the classification and prediction of data. To address the problem of datasets without vertex frame labeling, Liu et al. [27] used a lightweight network Squeeze Net to reliably locate the vertex frames in the dataset without vertex frame labels and applied a 3D convolutional network to extract spatio-temporal features. To address the problem of difficulty in focusing on the local details of micro-expressions, Zhao et al. [28] proposed the ME-PLAN deep learning framework to learn accurate micro-expression features through expression-related knowledge transfer and scenario training. Zhou et al. used a dual stream network to extract salient and discriminative features of facial expressions, thereby achieving micro expression recognition. Li et al. [29] proposed a deep fusion global feature extraction network for extracting global and local spatiotemporal features, finally achieving micro expression recognition. Zhao et al. [30] specially designed a six-layer CNN network to achieve the effective feature extraction of micro-expressions. Khor et al. [31] proposed a lightweight dual stream shallow network to improve the feature extraction network for feature extraction. Zhi et al. [32] concatenated CNN and LSTM to directly process micro-expression sequences of different time lengths, pioneering a new approach to micro-expression feature extraction.

Transformer is a deep neural network primarily based on the self-attention mechanism, initially applied in the field of natural language processing. The attention mechanism can allocate different weights to different features. Based on this characteristic, adding an attention mechanism in the channel and spatial dimensions can suppress background interference and enhance the network’s ability to perceive key features. Inspired by the powerful representation capabilities of Transformer, researchers have begun to propose the extension of Transformer to computer vision tasks. Ma et al. [33] first applied the Transformer architecture to expression recognition, in which the network first uses ResNet18 to extract the feature maps of the input image and finally puts them into a multilayer Transformer encoder for classification. Zhang et al. [34] proposed the SLSTT network; this network architecture feeds the micro-expression sequence optical flow features into the Transformer encoder and finally classifies them after the fusion of temporal and spatial features through the LSTM architecture. Zhongyang Liu et al. [35] fused multi-scale features based on the attention mechanism and demonstrated the effectiveness of multi-scale feature fusion for image classification. Che et al. [36] proposed a two-branch Transformer to extract different scale features and to fuse different branches based on a Cross Attention fusion mechanism. For the visual Transformer, the performance of the network can be effectively improved by improving the self-attention mechanism. Huang et al. [37] extended the traditional self-attention mechanism to determine the correlation between attention results and query results. Chunxia Yang et al. [38] proposed a model based on the fusion of BERT and the attention mechanism, showing that the Transformer architecture has better performance regarding the task of sentiment analysis. Inspired by the above literature, in this paper, the attention mechanism is improved to enhance micro-expression recognition accuracy.

## 3. Proposed Method

**Figure 1:**
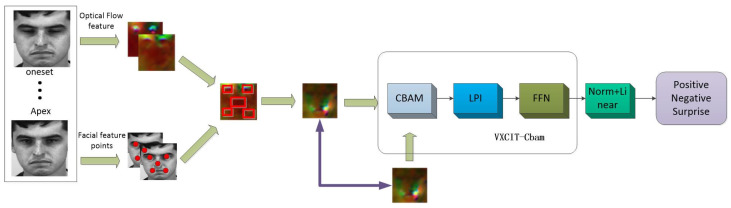
Architecture of MCCA-VNet micro-expression recognition model. Red box represents the important area of facial micro expressions.

The structure of the MCCA-VNet micro-expression recognition network proposed in this paper is shown in Figure 1. The feature extraction part is divided into four parts: data preprocessing, feature extraction, feature fusion, and classification. Image preprocessing includes face cropping, face alignment, apex frame screening, and other operations. Feature extraction includes optical flow feature extraction and facial feature point extraction, fusing the optical flow features in the key region through key point localization, effectively fusing the two features together, and, after obtaining the fused features, inputting them into the MCCA-VNet network and ultimately outputting the micro-expression recognition results.

### 3.1. Overall Architecture

**Figure 2:**
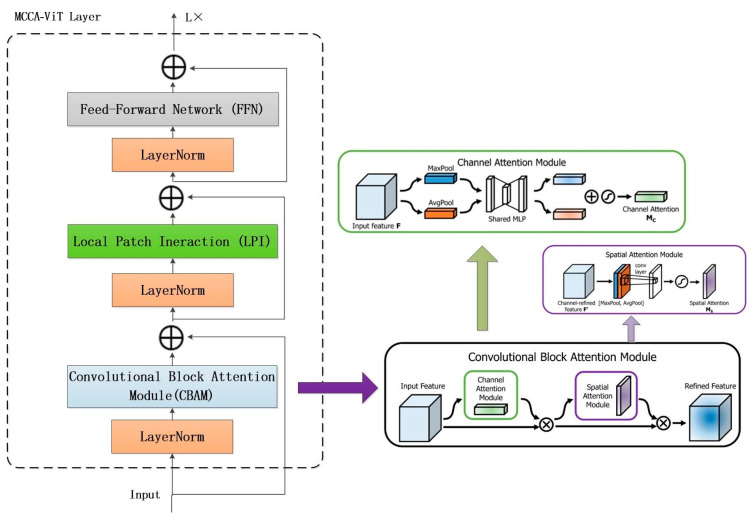
MCCA-VIT layer network structure diagram.

The overall framework structure of our network is based on the Vision Transformer (ViT) network framework and incorporates Cross-Covariance Image Transformers (XCIT) [39] and Convolutional Block Attention Module (CBAM) [40]; the innovation resulted in a new network, which we named MCCA-VNet (Multi Cross-Covariance Attention Vision Transformer Net). We use ViT as the original framework because it directly treats the image as a serialized input and utilizes the self-attention mechanism to deal with the pixel relationships in the image. ViT contains the self-attention mechanism and a feed-forward neural network layer so that contextual dependencies at different locations in the image can be captured and serialization can be achieved by classifying the output of the Transformer encoder. The classification of images considers the pixel dependencies and temporal correlations between different regions of the image for the micro-expression recognition task. We believe that ViT has high suitability for the micro-expression recognition task and apply it. See Figure 2.

### 3.2. Apex Frame Detection and Optical Flow Map Extraction

Optical flow maps generated from initial and apex frames as a method to characterize the motion of facial regions have been validated in various methods in the field of micro-expression recognition, and, in order to obtain the optical feature maps in such methods, the dataset needs to have the indexes of the initial and apex frames. However, some of the micro-expression datasets provide only the initial frame index without mentioning the apex frame index, which needs to be extracted from the video sequence by an algorithm. Unlike SAMM and CASMEII, which provide initial frame and peak frame indices, SMIC does not provide peak frame indices. In order to obtain the Apex frame indices of micro-expressions in the SMIC dataset, we employ the D & C-Rols method, which effectively establishes the relationship between the start frame and subsequent frames so that the apex frame index can be accurately identified, which ensures the reliable extraction of optical flow features for subsequent micro-expression recognition tasks.

We select the maximum value of the difference of LBP features as the apex frame, and this apex frame selection has been validated for effectiveness in micro-expression recognition methods.

(1) $$d=\frac{\sum_{i=1}^{B}h_{1i}\times h_{2i}}{\sqrt{\sum_{i=1}^{B}h_{1i}^{2}\times \sum_{i=2}^{B}h_{2i}^{2}}}$$

where *B* is the number of intervals in the grayscale histogram, which can be set by oneself; we divide 0–255 into 10 equal intervals, that is, *B* is set to 10, $h_{1i}$ is the grayscale histogram of the *i*-th interval in the first frame image, and $h_{2i}$ is the grayscale histogram of the i-th interval in the current frame. d is the highest value of the LBP feature difference rate between two frames.

After obtaining the apex frame index of the SMIC dataset, the categories of SMIC, SAMM, and CASMEII are merged and classified to form a mixed dataset with positive, negative, and surprising categories. This dataset has indices for initial and peak frames of micro-expressions. Then, the optical flow feature maps are obtained using the start and apex frames; since the TV-L1 optical flow estimation method is the most robust optical flow estimation method of all tests [41], we use it to compute the first and second channels of the input feature consisting of the horizontal component *u* and the vertical component *v*. In addition, we use them to compute the optical strain *ϵ*, which captures the subtle facial changes from the optical flow components.

(2) $$\epsilon =\left[\begin{array}{cc} \epsilon_{xx}=\frac{\delta u}{\delta x} & \epsilon_{xy}=\frac{1}{2}\left(\frac{\delta u}{\delta y}+\frac{\delta v}{\delta x}\right)\\ \epsilon_{yx}=\frac{1}{2}\left(\frac{\delta v}{\delta x}+\frac{\delta u}{\delta y}\right) & \epsilon_{yy}=\frac{\delta v}{\delta y} \end{array}\right]$$

$\epsilon_{xy}$ and $\epsilon_{yx}$ are the shear strain components, and $\epsilon_{xx}$ and $\epsilon_{yy}$ are the normal strain components. The third channel is the optical strain amplitude fed into the model |ϵ|, which can be denoted as:

(3) $$\left|\epsilon \right|=\sqrt{\epsilon_{xx}^{2}+\epsilon_{yy}^{2}+\epsilon_{xy}^{2}+\epsilon_{yx}^{2}}$$

In summary, [u,v,|ϵ|] form a three-dimensional optical flow feature map and are denoted as $V_{m}=\left[V_{x},V_{y},V_{z}\right]$ and $V\in R^{H\times W\times 3}$.

In our network, we used a region-specific processing approach to focus attention on five specific facial regions—the left eye region, the right eye region, the nose region, the left lip region, and the right lip region.

**Figure 3:**
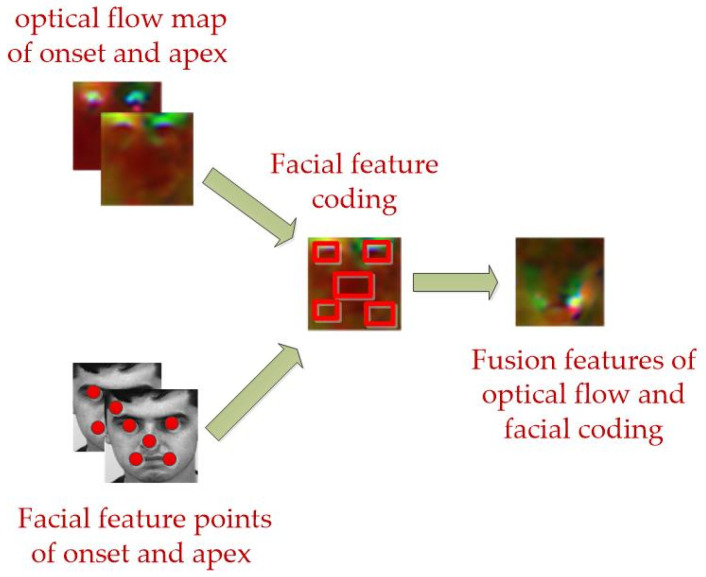
Light flow map extract fusion structure diagram.

First, we use (MTCNN) [42] to obtain the facial feature points from the micro-expression apex frames, and then we crop the region around the five facial feature points to obtain five facial optical flow maps, specifically, the optical flow map of the nose is centered on the nose feature point, and a certain number of pixel points on the left, right, top, and bottom are used as the feature regions of interest, which are cropped into the corresponding feature maps, and the eye region and lip region are similar to the nose region; after extracting the five optical flow field features, we combine them and input the combined features into our MCCA-VNet network. See Figure 3.

### 3.3. MCCA-Vit Layer Network Architecture

In our method, when processing the input optical flow image of size H × W × 3, the size of each facial image region block is inconsistent, and after linear projection and partitioning on the optical flow image, the corresponding feature regions are generated, and each region is recombined according to the facial localization to get the feature map of H × W, which is used as the input to the model, denoted as $\in {\mathbb{R}}^{b\times H\times W}$, where *b* is the batch size. The input feature map is cropped into corresponding patch blocks according to the size of the customized patches, after which the patches are fed into the linear embedding layer, and the corrected patches are next put into the transformer block. Multiple MCCA-Vit layers are applied to the transformer block, and the MCCA-Vit layer is borrowed from the VIT. The MCCA-Vit layer draws on the modeling ideas of VIT, Xcit, and CBAM, based on the implicit spatial and channel relationships inherent in micro-expressions, realizes explicit communication across patches by adding the covariate attention mechanism, so as to establish the connection between different patches and solve the problem of the non-interaction of information between different patches, realizes the feature enhancement of the channel attention and spatial attention information by adding CBAM, and integrates micro-expression features in different directions to achieve the accurate recognition of micro-expressions.

**Figure 4:**
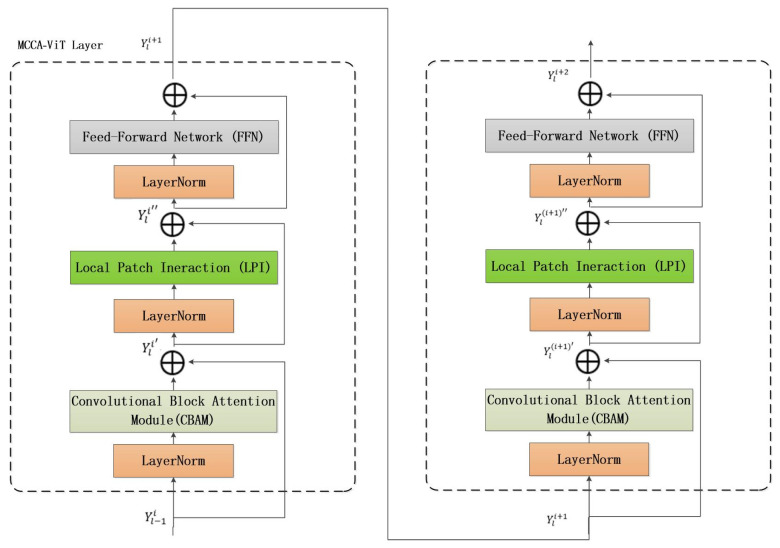
Schematic diagram of MCCA-Vit nested layers.

In order to encode the spatial information, trainable location embedding vectors are added to all the input sequence vectors, which ensures that the spatial relationship and location information are effectively captured and encoded in the feature representation, while multiple MCCA-Vit layers are nested to achieve multi-channel, multi-local module feature fusion and finally input to the standard output header to achieve micro-expression classification.

(4) $$Y_{l}^{i^{\prime }}=Y_{l-1}^{i}+CBAM\left(LN\left(Y_{l-1}^{i}\right)\right)$$

(5) $$Y_{l}^{i^{\prime \prime }}=Y_{l}^{i^{\prime }}+LPI\left(LN\left(Y_{l}^{i^{\prime }}\right)\right)$$

(6) $$Y_{l}^{i+1}=Y_{l}^{i^{\prime \prime }}+FFN\left(LN\left(Y_{l}^{i^{\prime \prime }}\right)\right)$$

(7) $$Y_{l}^{{\left(i+1\right)}^{\prime }}=Y_{l}^{i+1}+CBAM\left(LN\left(Y_{l}^{i+1}\right)\right)$$

(8) $$Y_{l}^{{\left(i+1\right)}^{\prime \prime }}=Y_{l}^{{\left(i+1\right)}^{\prime }}+LPI\left(LN\left(Y_{l}^{{\left(i+1\right)}^{\prime }}\right)\right)$$

(9) $$Y_{l}^{i+2}=Y_{l}^{{\left(i+1\right)}^{\prime \prime }}+FFN\left(LN\left(Y_{l}^{{\left(i+1\right)}^{\prime \prime }}\right)\right)$$

where *l* = 1, 2, …, *L* is the index of the lth block in each nested layer *I* and *L* is the total number of blocks in the nested structural layer (see Figure 4).

Each MCCA-Vit layer consists of a hybrid attention (CBAM) module, a local patch interaction (LPI) module, and a feed-forward network (FFN). CBAM contains two sub-modules, namely a channel attention module (CAM) and a spatial attention module (SAM); the CAM is mainly used to adaptively scale the feature map of each channel by calculating the importance of each channel, and the SAM is mainly concerned with the interdependence between different spatial locations. The CAM and SAM are connected serially, and they can focus on the key information in the feature map as shown in the following equation:

(10) $$F^{\prime }=M_{c}\left(F\right)⊗F,$$

(11) $$F^{\prime \prime }=M_{s}\left(F^{\prime }\right)⊗F^{\prime },$$

where *F* is the input feature and $F^{\prime }$ the features output after CAM, $F^{\prime \prime }$ is the feature output by SAM, $M_{c}$ is the CAM modeling algorithm, and $M_{s}$ is the SAM model algorithm, denoted as follows:

(12) $$M_{c}=\sigma \left(MLP\left(AvgPool\left(F\right)\right)+MLP\left(MaxPool\left(F\right)\right)\right)$$

(13) $$M_{s}=\sigma \left(f^{7\times 7}\left(AvgPool\left(F\right)\right)+MLP\left(MaxPool\left(F\right)\right)\right)$$

where σ is the sigmoid function and $f^{7\times 7}$ is a convolution kernel of size of the convolution kernel.

**Figure 5:**
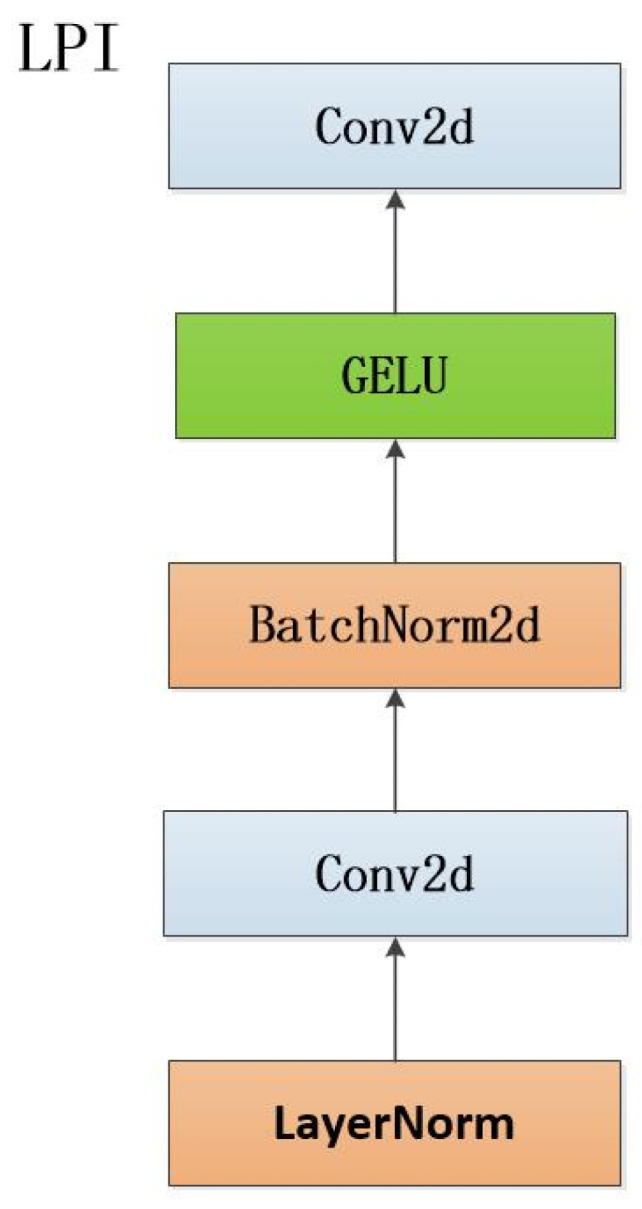
LPI net structure.

LPI contains different BatchNorm and GELU activation functions, which are nonlinearly transformed by Gaussian error function to enhance the expression and learning ability of the model and realize the explicit communication between cross-Patches, which ensures the input and output of features and the feature expression ability of the model in the middle layer. See Figure 5.

**Figure 6:**
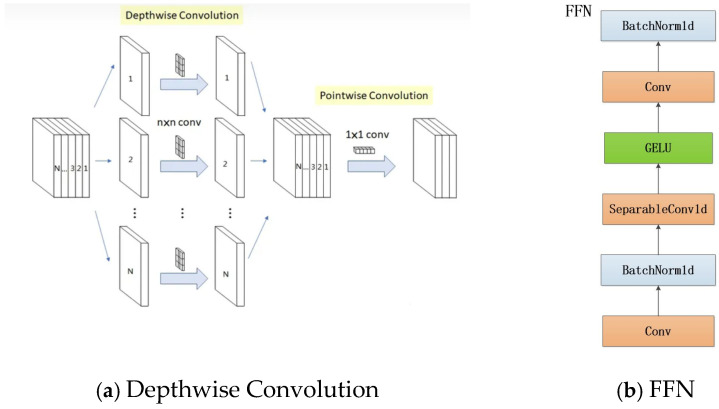
FFN net structure.

FFN has a single hidden layer with a four-dimensional hidden unit, which is used as an alternative to ordinary convolution through depth-separated convolutional layers, and the addition of activation function and BatchNorm after Depthwise Convolution helps to improve the nonlinear expression ability of the network and improve the computational efficiency in the network and fuses the feature maps between the channels, which simplifies the computation while ensuring the efficient extraction and fusion of micro-expression features. See Figure 6.

### 3.4. Loss Function Loss Function

In the loss function, the cross-entropy loss function can improve the accuracy of prediction. Our algorithm chooses a binary cross-entropy loss function for training micro expression recognition, and the specific loss function expression is as follows:

(14) $$\mathrm{loss}=-\frac{1}{N}\overset{M}{\underset{m=1}{\sum }}\overset{N}{\underset{n=1}{\sum }}{\hat{G}}_{mn}logO_{mn}$$

where *N* is the number of categories, *M* is the number of samples, ${\hat{G}}_{mn}$ is the sign function, taking 1 if the sample is correctly predicted and 0 otherwise, and $O_{mn}$ is the predicted probability that the sample belongs to category n.

## 4. Results

In this study, we conducted experiments on three databases: SAMM [43], SMIC [44], and CASMEII [45,46]. To ensure consistency and comparability, SAMM, SMIC, and CASMEII were combined into one comprehensive dataset, in which all three datasets used the same emotion labels for the micro-expression recognition task. In these datasets, the emotion categories are categorized as follows: the “positive” emotion category includes the “happy” emotion category, and the “negative” emotion category includes the “sad”, “disgust”, “fear”, and “anger” emotion categories. The category “surprise” is categorized separately.

### 4.1. Evaluation Datasets

#### 4.1.1. SAMM Dataset

The SAMM dataset includes 28 participants, 133 micro expressions, and 147 long videos containing 343 macro expressions. The resolution of the original images in this dataset is 2040 × 1088 pixels, with a frame rate of 200 frames per second. There are eight categories of emotions in SAMM images, namely “disgust”, “fear”, “contempt”, “anger”, “repression”, “surprise”, “happiness”, and “other”. After being categorized into “negative”, “positive”, and “surprised”, the three categories of “negative”, “positive”, and “surprised” are 92, 26, and 15, respectively. The SAMM dataset contains indexes of micro-expression start frames, end frames, and apex frames.

#### 4.1.2. CASMEII Dataset

The CASMEII dataset includes data from 24 subjects, totaling 145 samples corresponding to 145 emotions. All samples were captured with a laboratory video camera with the frame rate set to 200 frames per second. The original size of the samples was 640 × 480 pixels, and the samples in CASMEII were categorized as “happy”, “surprised”, “disgusted”, “sadness”, “fear”, “repression”, and “other”. The number of “negative”, “positive”, and “surprised” categories is 88, 32, and 25, respectively, and CASMEII contains indexes for the start, end, and apex frames of the micro-expressions. CASMEII contains an index of the start frame, end frame, and apex frame of the micro-expressions.

#### 4.1.3. SMIC-HS Dataset

The SMIC-HS dataset includes 16 subjects and 164 samples corresponding to 164 emotions. The frame rate is 100 frames per second. The original image size of the sample is 640 × 480 pixels. The SMIC dataset is classified into “negative”, “positive”, and “unexpected”, with numbers of 70, 43, and 51, respectively. SMIC provided the start and end frames of micro-expressions but did not provide an index of apex frames. Table 1 summarizes the detailed information of these three datasets.

### 4.2. Performance Metrics

In the composite micro-expression dataset we organized, there is a significant difference in the proportion of category distribution, with different emotions having varying proportions. The proportion of negative, positive, and surprising emotions is approximately 3:1.3:1. To address the issue of sample imbalance, we used non weighted average recall (UAR) and non-weighted average F1 score (UF1) to evaluate the results of micro expression recognition.

The unweighted F1 score (UF1) is a commonly used criterion for evaluating classification accuracy. We calculate UF1 by cross-counting the false positives (FP), true positives (TP), and false negatives (FN) for each category in all test results using the (LOSO) method; then, we can use the following formula to calculate the F1 score for each class and finally to find the total F1 score.

(15) $$F_{1c}=\frac{2\times TP_{c}}{2\times TP_{c}+FP_{c}+\times FN_{c}}$$

(16) $$\mathrm{UF}1=\frac{F_{1c}}{c}$$

In Equation (15), *c* is the number of categories.

Unweighted average recall (UAR) is a particularly useful metric for evaluating the effectiveness of a model in cases of imbalanced class proportions. UAR can be defined as follows:

(17) $$\mathrm{UAR}=\frac{1}{c}\underset{c}{\sum }\frac{TP_{c}}{n_{c}}$$

In Equation (17), $n_{c}$ is the total number of samples in each category.

### 4.3. Settings

The operating system used in our experiments is ubuntu18.04, the graphics card configuration is 2 NVIDIA RTX 3090 GPUs, the deep learning framework used is PyTorch based on python3.8, and, during training, Initialize settings are as follows: the epoch is set to 800, the batch size to 256, the optimizer type to Adam, the learning rate to 5 × 10^−5^, the input face size to 28 × 28, number of hiddens to 768, and classes to 3; training the network for every 800 epochs will take about two days.

First, our model needs to determine the micro-expression apex frames; there is no apex frame index in the SMIC dataset, and we used the D & C-Rols technique to determine the index of the apex frames. SAMM and CASMEII have apex frame indexes, and the micro-expression initial frames and apex frames can be obtained directly during image preprocessing. After acquiring the initial and apex frame images from the dataset, we extracted the optical flow from the images at these two time points using the TV-L1 optical flow estimation method. Next, the three elements of the optical flow image—horizontal, vertical, and optical strain elements—have 28 × 28 × 3 pixels. After that, we extract the region of interest locations from the apex frame image using a Multi-task Cascaded Convolutional Network (MTCNN). Based on the region of interest locations, we extracted the optical flow feature maps for five important facial regions, i.e., left eye, left lip, nose, right eye, and right lip. These five optical flow feature maps are combined to be the size of the entire optical flow image, with sizes of 14 × 10 × 3, 14 × 10 × 3, 28 × 8 × 3, 14 × 10 × 3, 14 × 10 × 3 pixels, respectively. By focusing on these specific facial regions, we can effectively capture the facial muscle movements associated with micro-expressions. After extracting and combining the five optical flow feature maps and inputting them into our MCCA-Vit model, we are finally able to recognize and classify micro-expressions accurately and efficiently.

### 4.4. Permormance

In Table 2, we show the performance of our proposed method with previous manual and deep learning methods on micro-expression datasets including CASMEII, SMIC, and SAMM. The evaluation metrics used are unweighted F1 score (UF1) and unweighted average recall (UAR). Bold text is used to highlight the best results of the methods in each dataset and metric, showing the quantitative evaluation results of our MCCA-VNet network compared to other classically excellent methods on the metrics of unweighted F1 score (UF1) and unweighted average recall (UAR). It can be observed that comparing the once optimal results of SLSTT-LSTM, our method improves UF1 by 5.7% and UAR by 8.0%, and our method outperforms HTNet in UF1 and UAR by 8.1% and 8.8%, respectively. We applied the algorithm XCIT-Vit to the field of micro-expression recognition and also achieved excellent results, with great improvement over the HTNet algorithm; compared with the direct application of XCIT-Vit to the micro-expression recognition method, our method improved 3.6% on UF1 and 4.3% on UAR. The results show that our method can accomplish the micro-expression recognition task more accurately.

### 4.5. Ablation Study

In this section, we conduct an extensive ablation experimental study to evaluate different network variants in order to analyze the factors affecting the network performance.

#### 4.5.1. Impact of Different Facial Encoding

The combined encoding of facial optical-flow maps helps in micro-expression recognition. The input to our model is a 28 × 28 facial coding optical-flow map, so the combination and design of different facial codes can be used as experimental conditions for the ablation experiments; a UF1 score of 0.8326 and a UAR score of 0.8146 were obtained on the composite dataset in the absence of nasal region features in our model, a UF1 score of 0.7411 and UAR score of 0.7326 were obtained on the SMIC, a UF1 score of 0.9587 and UAR score of 0.9545 on the CASMEII dataset, and a UF1 score of 0.8832 and UAR score of 0.8712 on the SAMM dataset. After fusing the designed nasal region features with the lip and eye region features, the model can improve the UF1 score and UAR score to 0.8676 and 0.8622 for the composite dataset, 0.8157 and 0.8106 for the SMIC dataset, 0.9151 and 0.9231 for the CASMEII dataset, and 0.8832 and 0.8712 for the SAMM dataset. The feature fusion of the four regions in the CASMEII dataset achieved better results, but the fused features in the combined dataset containing the nose region had the best micro-expression recognition results, which highlights that effective facial coding can help to improve micro-expression recognition ability. Based on this, the experiments in this thesis adopt the third of the following table coding method.

The coding structure is defined as follows (see Table 3 and Table 4):

#### 4.5.2. Impact of Different Number of Attention Heads

First, we conducted ablation experiments on the number of attentional heads in MCCA-VNet, keeping the other network structures constant and taking different numbers of attentional heads separately, and tested the effect of the number of heads in the MCCA-Vit layer on the accuracy of the composite datasets SMIC, SAMM, and CASMEII. The unweighted F1 score (UF1) (a) neutralized unweighted average recall (UAR) performance of the composite dataset is shown in Figure 5; it shows that the 12 heads in the MCCA-Vit layer will have the best performance.

The results of the experiment are as follows (see Table 5):

#### 4.5.3. Impact of Different Sizes of Kernel_Size

In the CBAM module in MCCA-VNet, the size of the convolution kernel for feature extraction is adjusted by setting kernel_size. In spatial attention, as shown in Table 6, the target feature extraction is better at large convolution kernels, and more global features can be extracted.

#### 4.5.4. Impact of Different Number of MCCA-VIT Blocks

First, we experimentally compared different MCCA-Vit module nesting layers on MCCA-VNet; the results of the ablation experiments are shown in Table 2. The effect of different nesting structures in the model is evaluated in the ablation study by the unweighted F1 score (UF1) and unweighted average recall (UAR) metrics on the composite dataset, which can accurately express the effect of different numbers of nesting structures on the accuracy of micro-expression recognition. Our model without the MCCA-Vit hierarchy can only obtain a UF1 score of 0.4152 and a UAR score of 0.4295 on the composite dataset, a UF1 score of 0.4601 and UAR score of 0.4868 on the SMIC dataset, a UF1 score of 0.3388 and UAR score of 0.3816 on the SAMM dataset, and a 0.3473 UF1 score and 0.3742 UAR score on the SAMM dataset. After using 12 MCCA-Vit layers, the model can improve the UF1 scores and UAR scores to 0.8676 and 0.8622, respectively, on the composite dataset, 0.8157 and 0.8106 on the SMIC dataset, 0.8157 and 0.8106 UF1 scores and UAR scores of 0.9607 and 0.9620 for the CASMEII dataset, and UF1 scores and UAR scores of 0.8832 and 0.8712, respectively, for the SAMM dataset. The optimal micro-expression recognition of the model was achieved with a total of 12 MCCA-Vit layers, which highlights the fact that the MCCA-Vit module will enhance the ability to accurately recognize micro-expressions. A different number of nested layers affects the performance of the MCCA-VNet network (see Table 7).

## 5. Discussion

**Figure 7:**
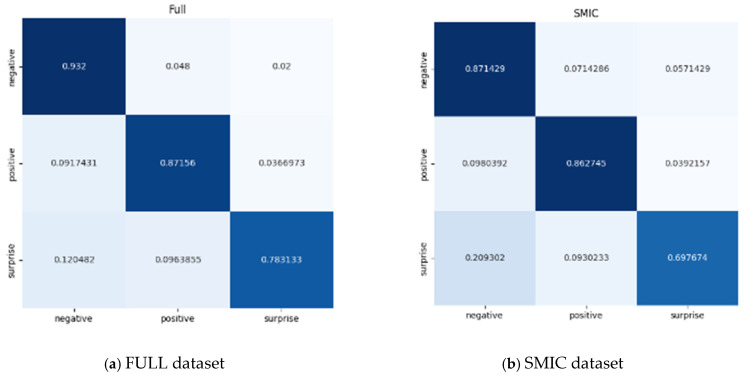

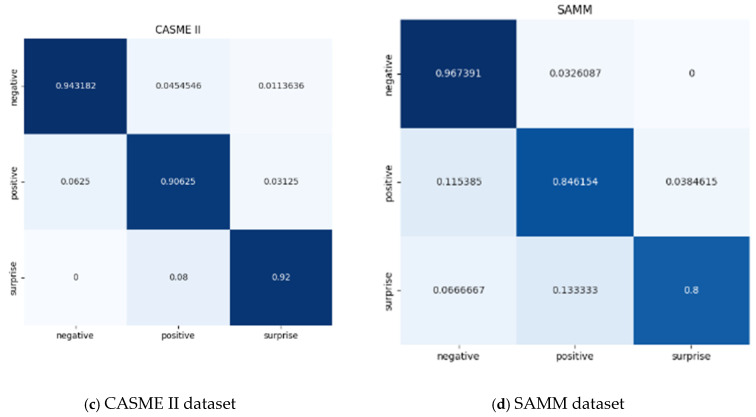
Confusion matrices for the proposed MCCA-ViT on the composite database (SMIC, SAMM, CASME II) using three classes. Different color’s background in the figure means the value of confusion matrices, the depth of color represents the magnitude of numerical values.

Figure 7 shows the confusion matrix of MCCA-VNet, demonstrating the accuracy of each sentiment category on the full composite dataset. In the composite dataset, the accuracy of MCCA-VNet is 0.932, 0.8756, and 0.783133 for the negative, positive, and surprise categories, respectively. In terms of lighting for dataset collection, the CASMEII, SAMM, and SMIC datasets were all collected in the laboratory. CASMEII and SAMM control the lighting to prevent image flickering, while the SMIC dataset has an infrared camera to capture facial images to prevent light interference. We use images captured by the SMIC visible light camera, so there may be lighting interference during micro expression recognition in the SMIC dataset. The accuracy of MCCA-VNet in the CASMEII dataset is 0.94318182, 0.90625, and 0.92 for the negative, positive, and surprise categories, respectively, which is more than 90% for each category with the least misclassification. It further validates the effectiveness of MCCA-VNet on this dataset. In addition, the SMIC dataset has more background noise, doped illumination changes, and unlocalized apex frames, which affect the accuracy of positive and unexpected emotion classification in the SMIC dataset, which are 86% and 69%, respectively. The accuracy of the negative, positive, and surprise categories in the SAMM dataset is 0.9673913, 0.84615385, and 0.8 respectively; the higher accuracy of the negative category can be attributed to the larger number of training samples for this category in the SAMM dataset, while the small number of training samples for the other two categories results in a slightly lower final recognition rate. It can be seen that in the field of micro-expression recognition, factors such as apex frame positioning, variations in illumination, etc. and imbalance problems between the sample categories in the dataset affect the recognition results, and it is extremely important to use appropriate methods for apex frame positioning and image preprocessing when performing micro-expression recognition.

According to the analysis of experimental results, our method achieves excellent results in comparison with other methods after image preprocessing and apex frame localization and achieves high recognition accuracy in different datasets, which is helpful for the next practical application.

## 6. Conclusions

In this paper, we propose a micro-expression recognition network, MCCA-ViT, which achieves superior test results. Benefiting from some achievements of deep learning in other fields, we designed a ViT-based network framework, which improves the micro-expression feature extraction capability and recognition accuracy by introducing a CBAM module for the deep spatial and inter-channel information fusion of facial optical flow features encoded in different regions. In addition, the MCCA-ViT model is based on the ViT architecture, which applies Transformer to the field of computer vision and emphasizes the spatial correlation between local regions of an image, which is suitable for the field of micro-expression recognition and can model the complex dependencies between different parts of a facial expression.

Although the MCCA-ViT model has achieved good micro-expression recognition results, there are still some problems, such as micro-expression apex frames being difficult to obtain, and factors such as changes in illumination, pose, and other environmental factors can affect the performance of the MCCA-ViT model. Therefore, the future work plan is to further study more accurate micro-expression apex frame extraction algorithms. On the basis of the optical flow method, the deep learning method Swin Transformer is used as the backbone to locate peak frames by analyzing optical flow features. Fixed intervals are used as sliding windows and input into the network to obtain the confidence level of each interval. The maximum confidence interval of the peak frame is then obtained, and the half-width position of the interval is the peak frame index. Better deep learning networks should be applied to study micro-expression recognition methods that are more robust in handling lighting and posture on the basis of image preprocessing and feature extraction studies.

## Figures and Tables

**Table 1:** Composite dataset.

Database	SAMM	CASME II	SMIC
Subjects	28	24	16
Samples	133	145	164
Frame rate	200	200	100
Negative	92	88	70
Positive	26	32	51
Surprise	15	25	43
Onset index	√	√	√
Offset index	√	√	√
Apex index	√	√	×

√ means the dataset include the index, × means the dataset doesn’t include the index.

**Table 2:** *F*1 and *UAR* performance of handcraft methods, deep learning methods and our MCCA-VNet method.

Approaches	Full	°	SMIC	°	CASME II	°	SAMM	°
	UF1	UAR	UF1	UAR	UF1	UAR	UF1	UAR
LBP-TOP [13]	0.5882	0.5785	0.2000	0.5280	0.7026	0.7429	0.3954	0.4102
Bi-WOOF [26]	0.6296	0.6227	0.5727	0.5829	0.7805	0.8026	0.5211	0.5139
AlexNet [17]	0.6933	0.7154	0.6201	0.6373	0.7994	0.8312	0.6104	0.6642
GoogLeNet [16]	0.5573	0.6049	0.5123	0.5511	0.5989	0.6414	0.5124	0.5992
VGG16 [15]	0.6425	0.6516	0.5800	0.5964	0.8166	0.8202	0.4870	0.4793
OFF-ApexNet [18]	0.7196	0.7096	0.6817	0.6695	0.8764	0.8681	0.5409	0.5392
STSTNet [47]	0.7353	0.7605	0.6801	0.7013	0.8382	0.8686	0.6588	0.6810
CapsuleNet [48]	0.6520	0.6506	0.5820	0.5877	0.7068	0.7018	0.6209	0.5989
Dual-Inception [49]	0.7322	0.7278	0.6645	0.6726	0.8621	0.8560	0.5868	0.5663
EMR [46]	0.7885	0.7824	0.7461	0.7530	0.8293	0.8209	0.7754	0.7152
RCN [50]	0.7432	0.7190	0.6326	0.6441	0.8512	0.8123	0.7601	0.6715
FeatRef [19]	0.7838	0.7832	0.7011	0.7083	0.8915	0.8873	0.7372	0.7155
SLSTT-LSTM [34]	0.816	0.790	0.740	0.720	0.901	0.885	0.715	0.643
HTNet [8]	0.7977	0.7824	0.722	0.7147	0.9053	0.8957	0.7455	0.7291
Xcit-Vit [39]	0.8326	0.8146	0.7411	0.7326	0.8120	0.7753	**0.9587**	**0.9545**
MCCA-Vit (ours)	**0.8626**	**0.8513**	**0.8209**	**0.8167**	**0.8185**	**0.7847**	0.9360	0.9299

**Table 3:** Different Facial Encoding.

Number	Left Eye	Left Lip	Nose	Right Eye	Right Lip
1	−7~+7 −7~+7	−7~+7 −7~+7	0	−7~+7 −7~+7	−7~+7 −7~+7
2	−7~+7 −3~+4	−7~+7 −3~+4	−14~+14 −7~+7	−7~+7 −3~+4	−7~+7 −3~+4
3	−7~+7 −5~+5	−7~+7 −5~+5	−14~+14 −4~+4	−7~+7 −5~+5	−7~+7 −5~+5

**Table 4:** *F*1 and *UAR* performance of different facial encoding.

Numbers of Encode		1	2	3
Full	UF1	0.8281	0.8484	**0.8676**
Full	UAR	0.8097	0.8426	**0.8622**
SMIC	UF1	0.7701	0.7793	**0.8157**
SMIC	UAR	0.7613	0.775	**0.8106**
SAMM	UF1	0.7703	0.8228	**0.8832**
SAMM	UAR	0.7366	0.8161	**0.8712**
CASME II	UF1	0.9176	**0.9402**	0.9151
CASME II	UAR	0.8995	**0.9374**	0.9231

**Table 5:** *F*1 and *UAR* performance of different number of heads.

Number of Heads		4	8	12	16	20
Full	UF1	0.8384	0.8285	**0.8676**	0.8481	0.8483
Full	UAR	0.8223	0.8135	**0.8622**	0.8364	0.8309
SMIC	UF1	0.7869	0.7655	**0.8157**	0.7753	0.7741
SMIC	UAR	0.7779	0.7559	**0.8106**	0.7679	0.7635
SAMM	UF1	0.802	0.8435	**0.8832**	0.8657	0.854
SAMM	UAR	0.7774	0.8199	**0.8712**	0.8603	0.8383
CASME II	UF1	0.9246	0.8848	0.9151	0.9269	**0.9302**
CASME II	UAR	0.9099	0.8786	**0.9231**	0.9108	0.9175

**Table 6:** *F*1 and *UAR* performance of SIZES OF KERNEL_SIZE.

Sizes of Kernel_Size		3 × 3	7 × 7
Full	UF1	0.8276	**0.8676**
Full	UAR	0.8129	**0.8622**
SMIC	UF1	0.7474	**0.8157**
SMIC	UAR	0.7382	**0.8106**
SAMM	UF1	0.8134	**0.8832**
SAMM	UAR	0.7885	**0.8712**

**Table 7:** *F*1 and *UAR* performance of DIFFERENT numbers of MCCA-Vit blocks.

Numbers of MCCA-Vit Blocks		0	4	8	9	10	11	12
Full	UF1	0.4152	0.8126	0.8269	0.8237	0.8344	0.8379	**0.8676**
Full	UAR	0.4295	0.7802	0.8085	0.816	0.8229	0.823	**0.8622**
SMIC	UF1	0.4601	0.7411	0.8085	0.7646	0.7644	0.7769	**0.8157**
SMIC	UAR	0.4868	0.7202	0.806	0.7685	0.7601	0.769	**0.8106**
SAMM	UF1	0.3473	0.8085	0.7438	0.7941	0.822	0.8028	**0.8832**
SAMM	UAR	0.3742	0.7718	0.6983	0.7498	0.7941	0.7789	**0.8712**
CASME II	UF1	0.3388	0.8957	0.8822	0.8999	**0.9265**	0.9209	0.9151
CASME II	UAR	0.3816	0.8758	0.8777	0.8861	0.913	0.907	**0.9231**

## Data Availability

Data are contained within the article.
